# Comparing Web-Based and Lab-Based Cognitive Assessment Using the Cambridge Neuropsychological Test Automated Battery: A Within-Subjects Counterbalanced Study

**DOI:** 10.2196/16792

**Published:** 2020-08-04

**Authors:** Rosa Backx, Caroline Skirrow, Pasquale Dente, Jennifer H Barnett, Francesca K Cormack

**Affiliations:** 1 Cambridge Cognition Ltd Cambridge United Kingdom; 2 School of Psychological Science University of Bristol Bristol United Kingdom; 3 Department of Psychiatry University of Cambridge Cambridge United Kingdom

**Keywords:** reliability, mobile health, neuropsychological tests, CANTAB, cognition

## Abstract

**Background:**

Computerized assessments are already used to derive accurate and reliable measures of cognitive function. Web-based cognitive assessment could improve the accessibility and flexibility of research and clinical assessment, widen participation, and promote research recruitment while simultaneously reducing costs. However, differences in context may influence task performance.

**Objective:**

This study aims to determine the comparability of an unsupervised, web-based administration of the Cambridge Neuropsychological Test Automated Battery (CANTAB) against a typical in-person lab-based assessment, using a within-subjects counterbalanced design. The study aims to test (1) reliability, quantifying the relationship between measurements across settings using correlational approaches; (2) equivalence, the extent to which test results in different settings produce similar overall results; and (3) agreement, by quantifying acceptable limits to bias and differences between measurement environments.

**Methods:**

A total of 51 healthy adults (32 women and 19 men; mean age 36.8, SD 15.6 years) completed 2 testing sessions, which were completed on average 1 week apart (SD 4.5 days). Assessments included equivalent tests of emotion recognition (emotion recognition task [ERT]), visual recognition (pattern recognition memory [PRM]), episodic memory (paired associate learning [PAL]), working memory and spatial planning (spatial working memory [SWM] and one touch stockings of Cambridge), and sustained attention (rapid visual information processing [RVP]). Participants were randomly allocated to one of the two groups, either assessed in-person in the laboratory first (n=33) or with unsupervised web-based assessments on their personal computing systems first (n=18). Performance indices (errors, correct trials, and response sensitivity) and median reaction times were extracted. Intraclass and bivariate correlations examined intersetting reliability, linear mixed models and Bayesian paired sample t tests tested for equivalence, and Bland-Altman plots examined agreement.

**Results:**

Intraclass correlation (ICC) coefficients ranged from ρ=0.23-0.67, with high correlations in 3 performance indices (from PAL, SWM, and RVP tasks; ρ≥0.60). High ICC values were also seen for reaction time measures from 2 tasks (PRM and ERT tasks; ρ≥0.60). However, reaction times were slower during web-based assessments, which undermined both equivalence and agreement for reaction time measures. Performance indices did not differ between assessment settings and generally showed satisfactory agreement.

**Conclusions:**

Our findings support the comparability of CANTAB performance indices (errors, correct trials, and response sensitivity) in unsupervised, web-based assessments with in-person and laboratory tests. Reaction times are not as easily translatable from in-person to web-based testing, likely due to variations in computer hardware. The results underline the importance of examining more than one index to ascertain comparability, as high correlations can present in the context of systematic differences, which are a product of differences between measurement environments. Further work is now needed to examine web-based assessments in clinical populations and in larger samples to improve sensitivity for detecting subtler differences between test settings.

## Introduction

Cognitive function is typically assessed during one-to-one administration of a neuropsychological test in a clinic or lab setting by a trained psychometrician [1]. However, in-person assessments entail significant costs, requiring employed and trained staff, as well as time and travel costs for personnel and participants [2]. These costs may limit their application and reduce resources for clinical and research activities, including patient care, optimizing power for research, and screening for clinical trials [3]. The requirement for one-to-one test administration may also limit participation to people who are willing and able to travel, making some communities underrepresented in clinical research (eg, individuals who are geographically isolated, nondrivers, physically disabled, and those suffering from agoraphobia or social phobias).

Computerized testing platforms and widespread access to fast and affordable internet has the potential to bring neuropsychological assessment into people’s homes [2-4]. Web-based neuropsychological assessments could help to meet increasing demands in clinical and cohort studies [3,5]: providing access to large samples, allowing fine-grained phenotyping of complex clinical conditions, facilitating access to patients and participants in remote areas or those with mobility problems, enhancing coordination of data collection across multiple sites, assisting in monitoring of patients with chronic or progressive neurological diseases, and enabling cost-effective screening for clinical trials.

Web-based automated assessments are inexpensive, are quick to conduct, and provide fewer restrictions on timing and location [2,5-7]. Evidence suggests that broadly targeted web-based assessments allow the recruitment of samples that are reasonably representative in terms of personality and adjustment characteristics and are more diverse than traditionally recruited samples in terms of geographical location, gender, and socioeconomic status [7]. Moreover, web-based assessments can reduce the cost of recruiting specialized samples or special interest groups [4,7].

However, the joint position paper for the American Academy of Clinical Neuropsychology and the National Academy of Neuropsychology [8] highlights the necessity of viewing unsupervised computer-based tests as new and different from those that are examiner administered, with adaptations of existing tests requiring equivalency or new normative data. Key differences between examiner-led and unsupervised computerized testing relate to 3 primary factors, which are likely to interact with task-specific characteristics (such as simplicity of the user interface, audibility and clarity of stimuli and instructions, type of response required, and how engaging and how difficult a task is) to influence task performance:

1. Examiner contact: Social demands created by the presence of an examiner may affect performance [9]; examiner contact allows for behavioral observations to assess comprehension, mental state and competency, motivation, and task engagement [8,10]; the examiner can also provide additional explanation regarding tasks where needed [11], and structured encouragement to support participant motivation.
2. Testing environment: While the testing environment can be kept constant in the laboratory, it is uncontrolled elsewhere [8,10]. There is little control over the location, timing, and likelihood of participant distraction in unsupervised testing.
3. Workstation: Differences in the performance of computer hardware, software, processing speed, and internet speed, as well as response input method (touch screen versus key stroke or mouse click), are likely to impact test measures, particularly those relating to response timing [12].

Despite the key differences outlined earlier, web-based assessments have proven to be powerful for identifying age-related changes in cognitive processes [13], thus providing reliable data for a longitudinal and quantitative genetic analysis [2,14]. Previous reports have usually shown moderate correlations between web-based cognitive assessments and paper-and-pencil test variants [1,15], and moderate-to-high correlations between parallel computerized test versions assessing a broad range of cognitive domains administered in the lab and at home, or in supervised and nonsupervised settings [16-19]. This suggests that web-based cognitive assessment may be considered a viable alternative to in-person assessment.

Here, we examine the comparability of unsupervised web-based tests completed at home against in-person lab-based assessment in selected tests from the Cambridge Neuropsychological Test Automated Battery (CANTAB). CANTAB is a widely used computerized assessment battery [20], published in over 2000 peer review papers [21], and is widely used in academic, clinical, and pharmacological research [22]. CANTAB tests include a suite of 19 cognitive assessments measuring aspects of cognitive functioning in different therapeutic areas, including attention and psychomotor speed, executive function, memory, and emotion and social cognition. Tasks can be used individually or as a battery to measure different aspects of cognitive function. CANTAB is usually administered under controlled settings in the presence of a trained researcher or clinician.

This study aimed to determine the comparability of unsupervised web-based assessment on CANTAB against a standard in-person assessment in a healthy adult population. The aim was to examine the consistency of assessment outcomes across these 2 settings, and by extension to inform whether web-based testing could be used as an alternative or as a complementary assessment method producing similar results. We selected 7 tests from CANTAB, which correspond to those most frequently used in academic and clinical research in the cognitive domain of interest.

For web-based testing to show acceptable comparability, we required assessments to (1) show high levels of intersetting reliability, that is, the reproducibility of measures across settings [23], (2) show equivalence with in-person tests, and (3) meet established thresholds for agreement. Given the results from previous research comparing online and in-person tests reviewed earlier, we expected test performance indices to show acceptable comparability. However, we expected reaction time measures to perform more poorly due to the variance introduced by computing software, hardware, and response method.

## Methods

### Power Analysis

This study was powered to detect moderate-to-high intraclass correlations (ICCs) and moderate-to-large differences in test performance between test settings.

Power calculations to detect ICCs indicating adequate reliability were completed using the R package ICC.Sample.Size [24,25], a statistical package based on the work of Zou et al [26]. Using thresholds for clinical significance developed by Cicchetti [27], the following interpretations were adopted for ICC coefficients (ρ): <0.4, poor reliability; 0.40-0.59, fair; 0.60-0.74, good; 0.75-1.00, excellent. This indicated that a sample of 18 was required to detect an ICC that is indicative of good reliability (ρ=0.60) at 80% power, with a two-tailed *α* of .05. A sample of 45 would provide adequate power to detect an ICC that is indicative of fair reliability (ρ=0.40).

The power to detect differences between testing platforms was examined using the program G*power 3 [28]. This indicated that detecting an effect size of 0.4, at 80% power (two-tailed *α* at .05), would require a sample of 52 in a paired sample test with normal distribution, and between 35 and 47 for the nonparametric equivalent, depending on the underlying distribution of data (laplace and logistic, respectively). An effect size of 0.4 has been reported as relatively typical within psychological sciences [29,30]. This study utilizes the Bayesian approach as an adjunct to our frequentist analysis to consider the strength of evidence in favor of both the alternative and null hypotheses and compare their probabilities [31].

### Participants

Participants were approached via fliers and advertisements posted on Facebook, targeting Cambridge, United Kingdom, and the immediate surrounding areas. These directed potential participants to a web-based screening questionnaire, administered via SurveyMonkey [32], through which participants provided basic demographic data (sex, age, and education level) and responses to questions probing eligibility for the study (exclusion criteria: history of dyslexia, concussion, head injury, neurological or psychiatric conditions, and nonfluent in English).

A total of 51 healthy adults were recruited into this study (32 women and 19 men), aged between 20 and 77 years, with a mean age of 36.8 (SD 15.6) years. Participants were highly educated, with 17.6% with school-level qualifications and 82.4% with university-level education, reflecting the demography of this region. All participants provided informed written consent to participate.

### Procedure

Participants were allocated to one of the two groups (in-person first or web-based first), through randomization at the time of recruitment. However, where necessary, allocation from randomization was overridden, where participant availability or laboratory space constricted the timing of assessments. The allocation of test sessions was as follows: in-person testing first for 33 participants and web-based assessment first for 18 participants. Test sessions were completed on average 1 week apart (mean 7.24, SD 4.5 days, range 1-25 days, with the majority [82% of tests] between 3 and 9 days), again with variation due to participant and laboratory availability.

In-person assessments were completed at Cambridge University, Cambridge, United Kingdom. Participants were seated in a quiet room and presented with CANTAB loaded onto an iPad (iPad 9.7, IOS operating system, [33]). The CANTAB test administration is fully automated, with on-screen text instructions and additional voiceover guidance for each task, explaining task goals and response requirements. For tests requiring training in addition to instruction (see *Measures*), training trials are incorporated within the automatic test administration. The transition from training to tests proceeds automatically, as do transitions between tests. Responses were logged via the touch screen. A trained psychometrician was present, whose role was to provide technical support where needed or additional instructions where required as well as to log observations (distraction or problems) during task performance.

Web-based assessments were completed via the CANTAB Connect web-based testing feature [34]. This delivered assessments which, from the viewpoint of the participant, were identical to those administered in-person, with the exception that they were administered at home and on personal computing systems. Web-based testing was enabled only on desktop or laptop computers, and not on touch screen devices. Responses were logged using mouse or trackpad clicks. Identical to in-person assessments, test administration was automated, with on-screen text instructions and additional voiceover guidance for each task, training incorporated into tasks where required, and automatic transitions between tests. Web-based CANTAB tests are designed to be resistant to low bandwidth by preloading or caching of data, allowing tests to be run in offline mode in testing locations where internet connectivity is poor. The application code is designed for cross-browser support and uses ubiquitous HTML and JavaScript features to support commonly used platforms. Extensive automated and manual tests are carried out to test functionality across browsers and ensure that the tests operate correctly and record accurate data.

Distraction during web-based assessment was documented with inbuilt programming to log if tasks were completed in full-screen mode, or if the participant tabbed to another browser window during the task. Participants were also asked at the end of the testing session if they were distracted during testing, although the nature of the distraction was not queried. These different forms of distraction were logged, but not differentiated, in the study database during data collection.

### Measures

**Figure 1:**
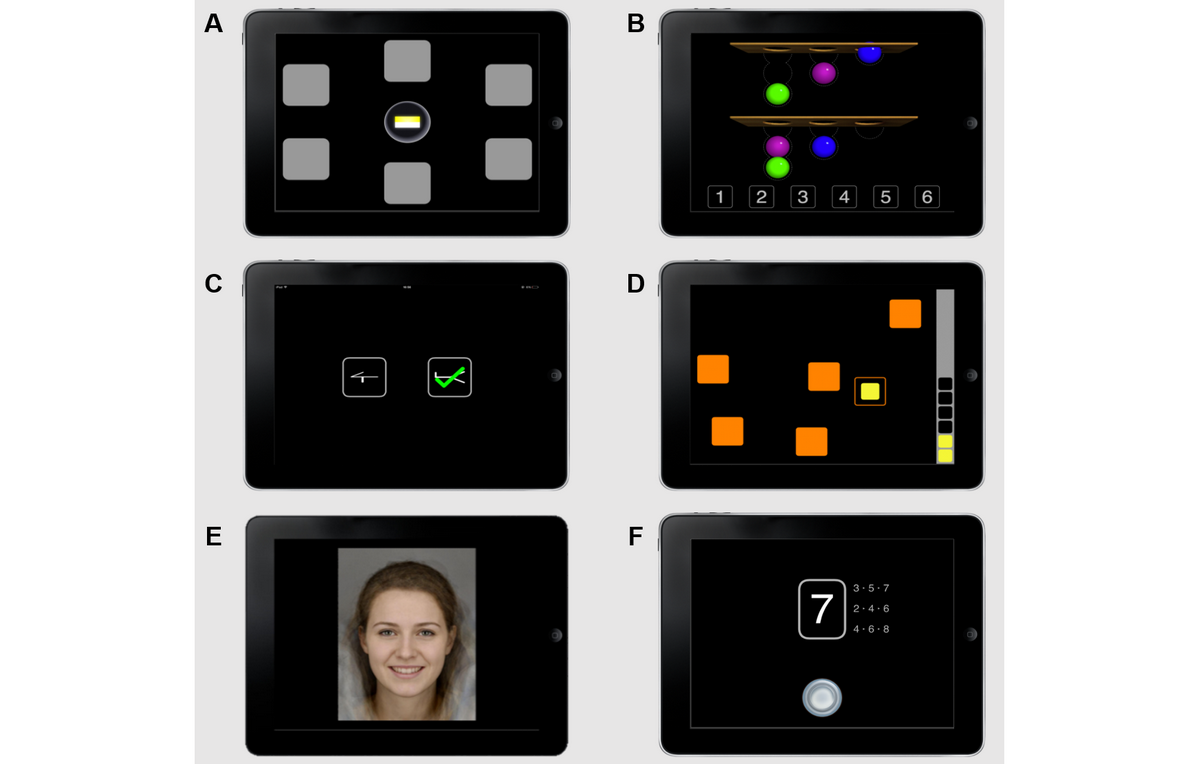
Screenshots of Cambridge Neuropsychological Test Automated Battery tests administered: (A) Paired Associate Learning, (B) One Touch Stockings of Cambridge, (C) Pattern Recognition Memory, (D) Spatial Working Memory, (E) Emotion Recognition Task, and (F) Rapid Visual Information Processing.

A total of 7 CANTAB tests (Figure 1) were administered. Cognitive outcome measures include performance indices (eg, number of trials solved, number of errors, response sensitivity) and reaction time (response times). For both in-person and web-based assessments, tests were administered in the following order:

1. Paired associate learning (PAL) [22] is an 8-min test of visual episodic memory. The screen displays a number of boxes and shows the interior of each box in randomized order to briefly reveal patterns in some boxes. Patterns are then displayed in the middle of the device screen one at a time, and the participant must identify the box in which each pattern was originally located. If an error is made, boxes are opened in sequence again to remind participants of the pattern locations. The test begins with a practice trial, which includes 6 boxes in which there are 2 patterns. Once the practice trial is successfully completed, the test begins. The task increased in difficulty after each successfully completed stage, with trials including 2, 4, and 6 different patterns in 6 boxes, and finally 8 different patterns in 8 boxes. The task discontinues when a participant fails to locate all patterns after 4 attempts on the same trial. Key outcome measures included PAL Total Errors Adjusted, the total number of errors adjusted for the stages not completed due to early discontinuation, and PAL First Attempt Memory Score, the number of times a participant chooses the correct box on their first attempt across each stage.
2. One touch stockings of Cambridge (OTS) [35] is a 10-min test of executive function, assessing spatial planning and working memory, and based on the Tower of London test. The screen shows 2 displays, each containing 3 colored balls that look like stacks held in stockings or socks suspended from a beam. The target configuration is shown at the top of the screen and the starting arrangement below. The subject must determine the number of moves required to match the starting configuration to the target. One move consists of taking 1 ball from its current location and placing it in a stocking that has free space. Only the top ball in any stocking may be moved (the balls below are inaccessible until any balls above have been moved), and a ball placed in a stocking drops to the lowest free space available. Participants must solve each problem without moving the balls, by indicating the number of moves required by selecting a numbered box at the bottom of the screen. The task begins with 3 training trials. The first two show how the balls would be moved before participants select their response, and the third only shows the solution when the participants’ response is incorrect. Once training is completed, the task then progresses with increasing difficulty. Key outcomes included problems solved on first choice and median latency to correct response.
3. Pattern recognition memory immediate (PRM-I) [36] is a 3-min test of immediate visual pattern recognition. A series of 18 simple but abstract stimulus patterns are shown in the center of the screen for 3000 ms each. The screen then displays pairs of patterns, one novel pattern and one that was shown previously. The participants have to select patterns that they recognize from the presentation phase. Participants receive performance feedback in the form of a tick or cross after every response. Key outcome variables include the percentage of correct responses and median latency of correct responses.
4. Spatial working memory (SWM) [35] is a 4-min test of retention and manipulation of visuospatial information. Participants click on colored boxes presented on the screen to inspect their contents and reveal a token hidden below. They then move these tokens to a collection area on the right-hand side of the screen. The key task instruction is that tokens will not be located in the same box twice during each trial. Outcome measures include SWM Between Errors: the number of times the participant incorrectly revisits a box, calculated across all assessed 4, 6, and 8 token trials; and SWM Strategy: the number of unique boxes from which a participant starts a new search in the 6 and 8 box trials. More efficient searches are carried out by searching boxes in a fixed order [37]. The task discontinues after 20 failed inspections during 4-token trials, 30 failed inspections for 6-token trials, and 40 failed inspections for 8-token trials.
5. The emotion recognition task (ERT) [38] is a 7-min test measuring participants’ ability to identify 6 basic facial emotion expressions along a continuum of expression magnitude. Participants fixate on a white “+” cross in the center of the screen for 1500 to 2500 ms, after which a face stimulus is displayed for 200 ms followed by a stimulus mask image for 250 ms. Participants then choose the most appropriate emotion from a list of 6 options (sadness, happiness, fear, anger, disgust, or surprise). Outcome measures included the total number of hits and median latency to correct responses.
6. Pattern recognition memory delayed (PRM-D) is a 2-min test of delayed visual pattern recognition. Patterns displayed for PRM-I are revisited and recognition is probed in the same manner as described in (3) after delay. In this study, the delay between PRM-I and PRM-D was approximately 12 min. Key outcome variables include the percentage of correct responses and median latency of correct responses.
7. Rapid visual information processing [39] (RVP) is a test of sustained attention lasting 7 min. Digits from 2 to 9 are presented successively at the rate of 100 digits per minute and in a pseudorandom order. Participants are asked to respond to target sequences of digits (eg, 3-5-7, 2-4-6, 4-6-8) as quickly as possible by clicking or pressing a button at the center of the device screen. The level of difficulty varies with either 1- or 3-target sequences that the participant must watch for at the same time. Outcome measures included a signal detection measure of response sensitivity to the target, regardless of response tendency (RVP A’: expected range is 0-1) and the median response latency.

CANTAB test structures are identical for each administration, across both in-person and web-based assessments. However, for most CANTAB tests (OTS, PAL, RVP, PRM, and ERT), stimuli are allocated at random from a broader stimulus pool during each assessment, making it unlikely that participants complete the same problems more than once. For the SWM test, token locations are not fixed but instead programmed to respond to participants’ performance and selection strategy, reducing the risk of participants being able to learn the location of tokens from one assessment to the next. These adaptive features aim to reduce practice effects on repeat testing and also mean that there are no set variants of the tests that can be compared in a group-wise fashion.

### Statistical Analysis

Frequentist analyses including mixed models, regressions, correlational analysis, and ICCs were completed in SAS version 9.4. Statistical significance thresholds were set at *P*≤.05 (two tailed). The Bayesian statistical analysis was carried out using JASP [40].

Outliers were identified using the methods recommended by Aguinis et al [41], first through visual plotting and then confirmed numerically, using a cutoff of 2.24 SD units above or below the mean. One data point was excluded from each of the following assessments: RVPA, RVP Median Latency to Correct Response, PRM Percentage Correct Immediate, and PRM Median Latency Immediate and Delayed (ranging 4.5-6.9 SD units from mean, all acquired during the web-based assessment).

To allow the comparison with test-retest reliabilities commonly reported in the literature [3,5,18,42], bivariate coefficients were computed to measure the strength of the linear association of outcome measures across test settings. Spearman rank correlations are reported because of the nonnormal distribution of data. To control for variation in the duration between assessments, partial correlations were completed, which examined correlations of test results between settings after covarying for the duration between tests.

However, although the correlational analysis reflects the degree to which paired observations follow a straight line, they do not inform regarding the slope of the line or whether the sets of observations capture the same metric or range of scores [43]. ICCs were selected as the primary reliability measure, because ICCs assume that the variables investigated share both their metric and variance and incorporate both random and systematic errors when calculating consistency between assessments [44,45]. ICCs therefore account for both consistency in performance (the degree of correlation) between test settings as well as capturing any systematic changes in the mean (the degree of agreement) [46]. Following guidance by Koo and Li [46] and justifications outlined in detail in Hansen et al [5], ICC was calculated based on a single-rating, absolute agreement, two-way random effects model (ICC 2,1 [47]). ICC coefficients were computed using the %INTRACC macro for SAS [48]. In line with previous studies and interpretative recommendations for ICC, we used ρ≥.60 to indicate good reliability [18,27].

Mixed effects models simultaneously investigated differences between the test settings (in-person vs web-based) and time (first vs second assessment). Mixed effects models can evaluate multiple factors that affect the structure of the data and allow longitudinal effects (practice and learning effects) to be straightforwardly incorporated into the statistical model [49]. Outcome measures were entered individually into each model as dependent variables, and 2 mixed effects models were analyzed for each outcome measure. The first model examined only the fixed effects of test setting and time of assessment, with participants entered into the model as a random effect. A second model was used to examine the presence of covariates that may affect test performance across settings, and included additional fixed effects of age, an age-by-setting interaction, and distraction during web-based testing (dummy coded as 1=distracted, 0=not distracted). This second model tested whether age affected performance and interacted with assessment setting to affect test results, and whether distraction during web-based assessment contributed to differences in test results.

The normality of the distribution of residuals was examined, and where required data were transformed before data analysis. Transformations included log transformations for PAL Total Errors Adjusted, SWM Between Errors, OTS Problems Solved on First Choice, and OTS Median Latency to Correct response and square root transformation for PAL First Attempt Memory Score. For most variables, transformations were successful and a linear mixed model was carried out (SAS command PROC MIXED). For PRM-I and PRM-D percentage correct, transformations were not successful. These data were reverse transformed (calculated as the percentage correct subtracted from 100) and were analyzed with mixed models with gamma error distributions and log links (SAS command PROC GLIMMIX).

Evidence in favor of the null hypothesis was examined using a Bayesian approach [50]. The advantage of using the Bayes factor over classical significance testing is that it provides a comparison of how likely the null hypothesis is compared with the alternative hypothesis [31]. Bayesian paired samples *t* tests were conducted, and Bayes factor test statistics were extracted, alongside effect sizes (*δ*) and their 95% credible intervals, contrasting the likelihood of data fitting under the null hypothesis (*H*_0_: no difference between test settings) with the alternate hypothesis (*H*_1_: that there is a difference between test settings). A default Chauchy prior width of *r*=0.707 was selected, and a Bayes factor robustness check was completed to examine if the qualitative conclusions changed with reasonable variations to the prior width. Bayes factors (BF_10_) were interpreted using a classification scheme adopted from Wagenmakers et al [51]: with Bayes factors below 1 seen as evidence for the null hypothesis (0.33-1: anecdotal evidence; 0.1-0.33: moderate evidence; <0.1 strong evidence for *H*_0_), and Bayes factors above 1 seen as evidence for *H*_1_.

Agreement between test settings was examined with Bland-Altman plots [52]. These plot the difference between assessments (eg, *A*−*B*) versus the average across paired measures (*A*+*B*/2), along with 95% limits of agreement [53]. The plots serve as a visual check that the magnitude of the differences is comparable throughout the range of measurement. Distributions of difference scores were assessed using Kruskal-Wallis tests, and where these were nonnormally distributed, raw data were log transformed before plotting and analysis. Other transformations were not considered, as these are not advised for this method of analysis [52,54]. Agreement is considered adequate when 95% of data points lie within limits of agreement [52]. Proportional bias was examined by regressing difference scores against mean scores to identify the tendency for the difference to increase or decrease with higher score magnitudes [55].

## Results

### Test Completion

Full test data were obtained from all participants with the exception of 2 individuals for whom the SWM test terminated early due a large number of errors made during web-based assessment. During in-person assessments, support from the examiner was required on 4 occasions (3 times for volume adjustment during PAL testing and once for additional instruction on the PRM immediate recognition task). Distraction, either through self-report or due to participants tabbing away from the assessment window during web-based assessments, was noted for 16 participants for PAL, ERT, OTS, and PRM-I tests and for 17 participants during SWM, RVP, and PRM-D tests.

### Reliability

Bivariate correlation coefficients and ICCs are shown in Table 1. Spearman correlation coefficients across testing settings ranged from 0.39 to 0.73 (*P*<.01). ICCs ranged from 0.23 to 0.67 (*P*≤.05). A total of 5 tests had ICC coefficients meeting the cutoff at ≥0.60, with PAL Total Errors Adjusted just meeting requirements (exact ICC coefficient=0.595, rounded up), and above threshold coefficients for RVP A’, SWM Between Errors, PRM-I Median Latency, and ERT Median Correct Reaction Time. Partial correlations of test results across settings after controlling for the duration between tests produced very similar results. These are shown in Multimedia Appendix 1.

### Equivalence

Descriptive statistics and results from the mixed model assessing fixed effects of test setting and time are presented alongside the Bayesian analysis results in Table 2. Mixed models revealed no significant differences between in-person and web-based assessments for performance indices (*P*=.10 to .54). However, 3 of the 5 reaction time measures showed differences across test settings (response latencies for PRM-I, PRM-D, and ERT tasks), with web-based assessments yielding slower median response times (*P*<.001 to .03). Practice effects were seen for RVP and SWM performance indices, showing improvement on second administration (*P*<.01). Response latencies were faster on the second administration for OTS responses (*P*=.001).

Additional fixed effects of age, an age-by-setting interaction effect, and distraction were incorporated into mixed models. Age effects on test performance, showing a decline in test performance with increasing age, were found for all outcome measures with the exception of RVP A’, the percentage of correct responses on PRM-I and PRM-D, and OTS Problems Solved on First Choice. No significant age-by-setting interactions were observed, indicating that test performance did not differ between in-person and web-based testing as a function of age, although there was a trend for slower reaction times on web-based testing for older participants on the PRM-I task (PRM-I Median Latency: *F*_1,45_=4.01, *P*=.051; for all other tests *F* statistic range 0.02-2.49; *P*=.12 to .90). Effects of distraction were nonsignificant for most tests, but reached or neared significance thresholds for certain reaction time measures (ERT Median Correct Reaction Time: *F*_1,47_=6.03, *P*=.02; RVP Median Reaction Time: *F*_1,46_=3.78, *P*=.06).

Bayesian analyses supported the null hypothesis (*H*_0_: no difference between test settings) over the alternate hypothesis: BF_10_=0.161-0.54) for all performance indices. Applying the classification scheme adopted from Wagenmakers et al [51], support for the null hypothesis was anecdotal for 3 variables (PAL First Attempt Memory Score, SWM Strategy, and ERT Total Hits), and moderate for 6 other performance indices. No change in the qualitative conclusions was seen with reasonable variations in the prior width. The effect sizes were small (0.15-0.27).

The alternate hypothesis, reflecting a difference between test settings, was supported for 3 out of the 5 reaction time measures (response latencies on PRM-I, PRM-D, and ERT tasks), with support being between anecdotal for the PRM measures (BF_10_=1.60-2.15) and very strong for ERT (BF_10_=512557.32). Effect sizes were in the low-to-large range (0.04-1.69). Moderate support for the null hypothesis was seen for the RVP and OTS reaction time measures.

**Table 1:** Reliability analysis for outcome measures of Spearman correlation coefficients and intraclass correlations between test results obtained in-person and in web-based assessments.

Outcome variable	Spearman correlation		Intraclass correlation	
	Correlation coefficient	*P* value	Correlation coefficient	*P* value
PAL^a^ total errors adjusted	0.54	<.001	0.60	<.001
PAL first attempt memory score	0.45	.001	0.51	<.001
OTS^b^ problems solved on first choice	0.39	.005	0.40	.002
OTS median latency to correct	0.55	<.001	0.45	<.001
PRM-I^c^ percentage of correct trials	0.40	.004	0.34	.008
PRM-I median latency	0.61	<.001	0.65	<.001
SWM^d^ between errors	0.61	<.001	0.62	<.001
SWM strategy	0.50	<.001	0.49	<.001
ERT^e^ total hits	0.54	<.001	0.57	<.001
ERT median correct reaction time	0.73	<.001	0.61	<.001
PRM-D^f^ percentage of correct trials	0.49	<.001	0.49	<.001
PRM-D median latency	0.57	<.001	0.56	<.001
RVP^g^ A’	0.71	<.001	0.67	<.001
RVP median latency	0.41	.003	0.23	.048

^a^PAL: paired associate learning.

^b^OTS: one touch stockings of Cambridge.

^c^PRM-I: pattern recognition memory immediate.

^d^SWM: spatial working memory.

^e^ERT: emotion recognition task.

^f^PRM-D: pattern recognition memory delayed.

^g^RVP: rapid visual information processing.

**Table 2:** Descriptive data for outcome variables and statistical results for equivalence analyses. Time at assessment (first vs second assessment) and test setting (in-person or web-based). Mixed effects model and Bayesian t test statistics

Outcome variable	Descriptive statistics					Mixed model test statistics						Bayesian paired *t* test statistics		
	Time of assessment, mean (SD)		Test setting, mean (SD)			First vs second assessment			In person vs web based			In person vs web based		
	First assessment	Second assessment		In-person	Web-based		*F* test (*df*)	*P* value		*F* test (*df*)	*P* value		Bayes factor *H*_1_	Effect size *δ* (95% Credible Intervals)
PAL^a^ total errors adjusted	12.06 (13.76)	11.00 (13.21)		12.43 (14.53)	10.63 (12.36)		0.12 (1,49)	.73		0.99 (1,49)	.33		0.259	−0.19 (−0.58 to 0.18)
PAL first attempt memory score	14.49 (4.31)	14.57 (3.83)		14.14 (4.28)	14.92 (3.81)		0.25 (1,49)	.62		1.87 (1,49)	.18		0.383	0.25 (−0.13 to 0.63)
OTS^b^ problems solved on first choice	11.73 (1.81)	11.90 (1.95)		11.69 (1.96)	11.94 (1.79)		0.06 (1,49)	.81		0.70 (1,49)	.41		0.221	0.15 (−0.21 to 0.53)
OTS median latency to correct (ms)	13933.22 (8130.39)	11525.04 (6651.39)		12718.31 (8124.91)	12764.24 (6878.99)		11.50 (1,49)	.001		2.18 (1,49)	.15		0.153	0.01 (−0.36 to 0.39)
PRM-I^c^ percentage correct	92.48 (13.67)	92.17 (12.76)		92.98 (11.83)	91.77 (14.48)		0.09 (1,37)	.77		0.91 (1,37)	.35		0.290	0.18 (−0.56 to 0.18)
PRM-I median latency (ms)	1533.67 (367.19)	1587.89 (448.00)		1506.80 (376.83)	1615.29 (434.55)		0.25 (1,48)	.62		4.36 (1,48)	.04		1.60	0.41 (0.04 to 0.80)
SWM^d^ between errors	7.80 (8.08)	4.92 (6.40)		6.96 (7.27)	5.82 (7.59)		7.59 (1,47)	.008		1.15 (1,47)	.29		0.229	0.16 (−0.55 to 0.20)
SWM strategy	7.04 (2.55)	5.67 (2.68)		6.71 (2.65)	6.02 (2.72)		12.50 (1,47)	<.001		0.71 (1,47)	.40		0.479	0.27 (−0.65 to 0.10)
ERT^e^ total hits	30.67 (4.18)	30.53 (4.52)		31.06 (4.13)	30.14 (4.5)		0.06 (1,49)	.80		2.78 (1,49)	.10		0.54	0.29 (−0.67 to 0.06)
ERT median correct reaction time (ms)	1274.50 (414.45)	1370.55 (510.74)		1174.45 (419.26)	1470.60 (465.65)		0.04 (1,49)	.83		39.79 (1,49)	.001		512557.32	1.25 (0.82 to 1.69)
PRM-D^f^ percentage correct	89.87 (12.73)	88.83 (15.49)		89.87 (14.17)	88.07 (14.14)		1.44 (1,31.2)	.24		0.63 (1,31.2)	.43		0.217	0.15 (−0.52 to 0.22)
PRM-D median latency (ms)	1731.65 (417.72)	1801.47 (463.71)		1698.15 (462.43)	1835.64 (409.44)		0.32 (1,48)	.58		4.90 (1,48)	.03		2.15	0.44 (0.05 to 0.83)
RVP^g^ A’	0.92 (0.05)	0.95 (0.04)		0.94 (0.04)	0.94 (0.05)		29.29 (1,48)	<.001		0.38 (1,48)	.54		0.161	0.17 (−0.21 to 0.54)
RVP median latency (ms)	452.96 (84.52)	436.98 (71.60)		449.32 (72.05)	440.37 (84.66)		0.97 (1,48)	.32		0.31 (1,48)	.58		0.183	−0.11 (−0.47 to 0.27)

^a^PAL: paired associate learning.

^b^OTS: one touch stockings of Cambridge.

^c^PRM-I: pattern recognition memory immediate.

^d^SWM: spatial working memory.

^e^ERT: emotion recognition task.

^f^PRM-D: pattern recognition memory delayed.

^g^RVP: rapid visual information processing.

### Agreement

**Figure 2:**
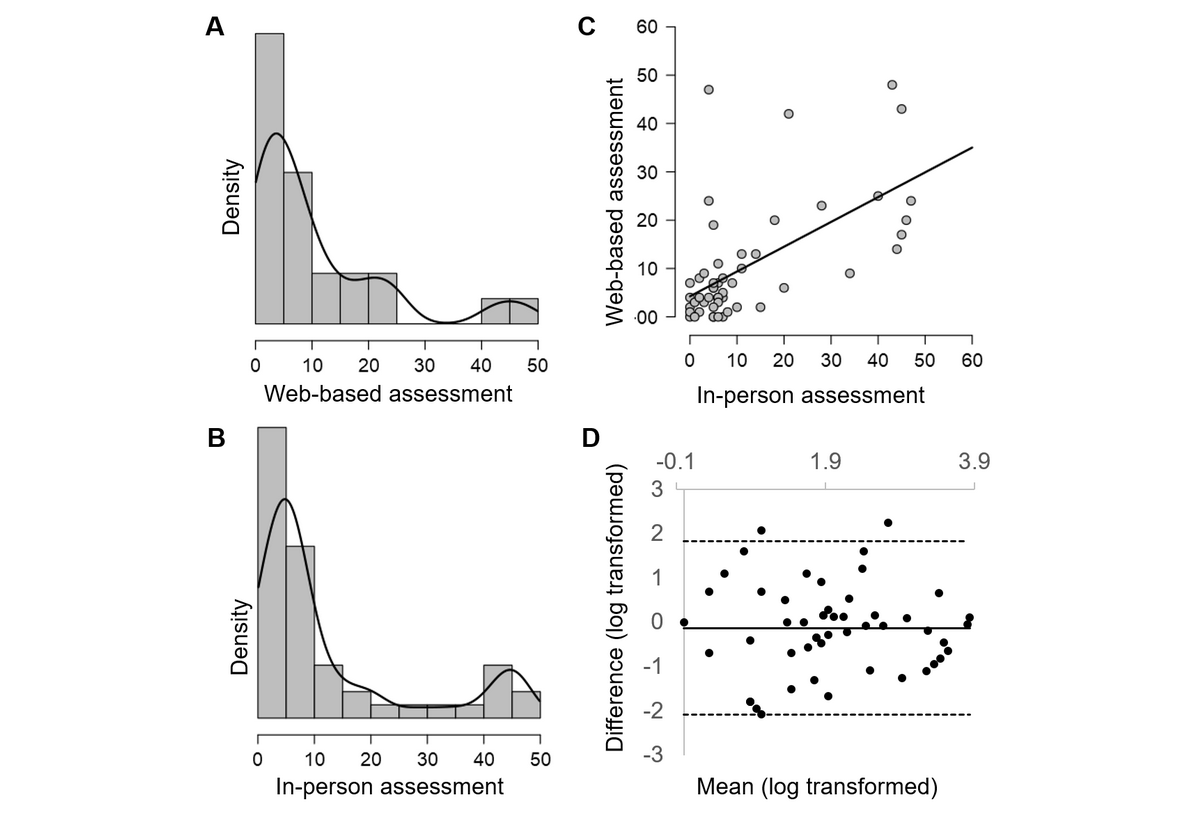
Comparability of Paired Associate Learning Total Errors Adjusted across test settings. Density plot for (A) web-based assessment and (B) in-person assessment showing similar distributions; (C) scatterplot with reference line showing linear relationship between assessment settings (ρ=0.54); (D) Bland-Altman plot: mean difference (solid black line) is close to zero, showing no bias; dashed lines delimit limits of agreement. Comparable magnitudes of difference are seen throughout the range of measurements, and 96% of the data within limits of agreement.

**Figure 3:**
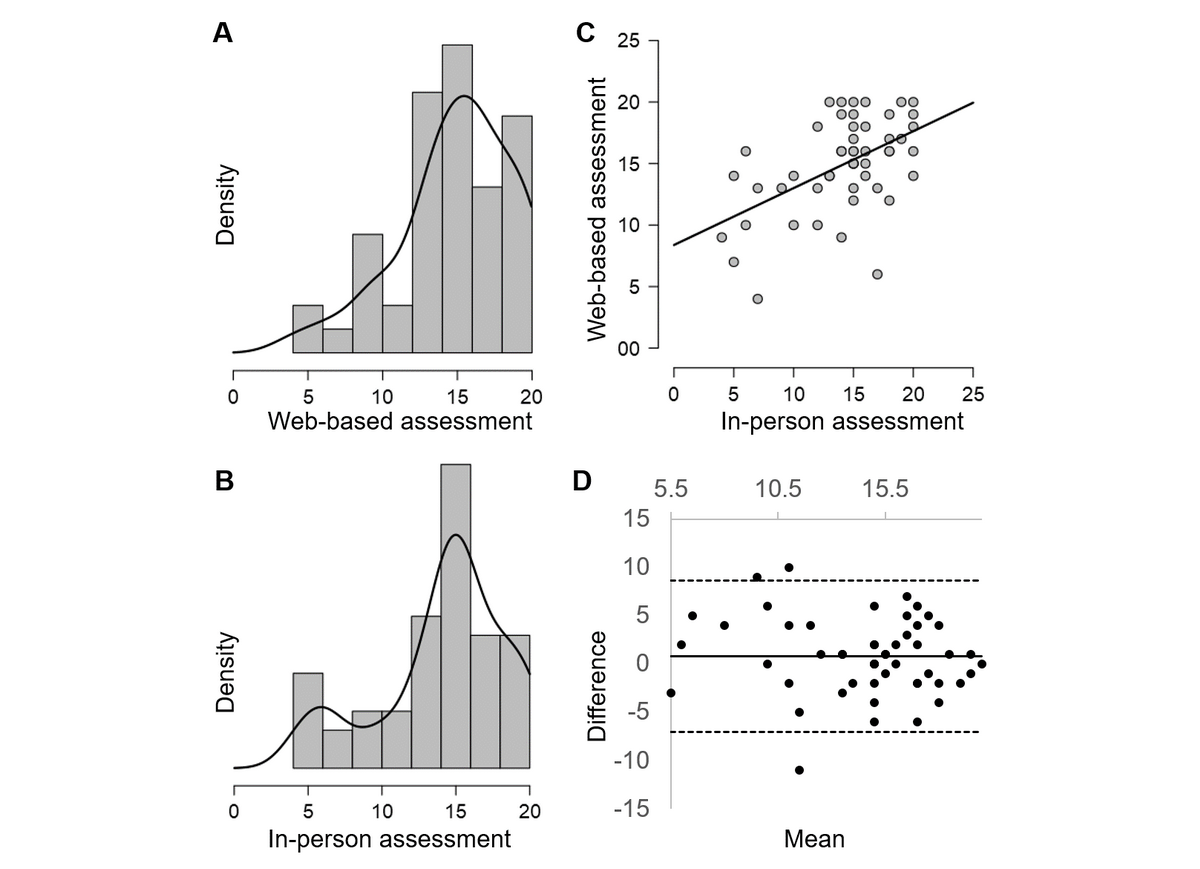
Comparison of Paired Associate Learning First Attempt Memory Score across test settings. Density plot for (A) web-based assessment and (B) in-person assessment showing similar distributions; (C) scatterplot with reference line showing linear relationship between assessment settings (ρ=0.45); (D) Bland-Altman plot: mean difference (solid black line) is close to zero, showing no bias; dashed lines delimit limits of agreement. Proportional bias is seen: greater differences at lower mean measurements and 94% of data within limits of agreement.

Bland-Altman plots showed overall good agreement between test settings for performance indices (see Figure 2, for example, for PAL Total Errors Adjusted). Only 2 performance indices fell short of the requirement that 95% of the data points should lie within limits of agreement (PAL First Attempt Memory Score and SWM Strategy, with 94% and 92% of data points within limits of agreement, respectively). The PAL First Attempt Memory Score showed a proportional bias (*F*_1,50_=7.43; *P*=.009; *R*^2^=0.13), with lower mean scores being associated with greater difference between measurements (Figure 3). For all other performance measure plots, no bias was seen relating to the test setting, and difference magnitudes were comparable throughout the range of measurements. Performance data from PRM tasks and from SWM Between Errors could not be accurately visualized using Bland-Altman plots because of significant nonnormality of the difference scores that could not be corrected through logarithmic transformation.

**Figure 4:**
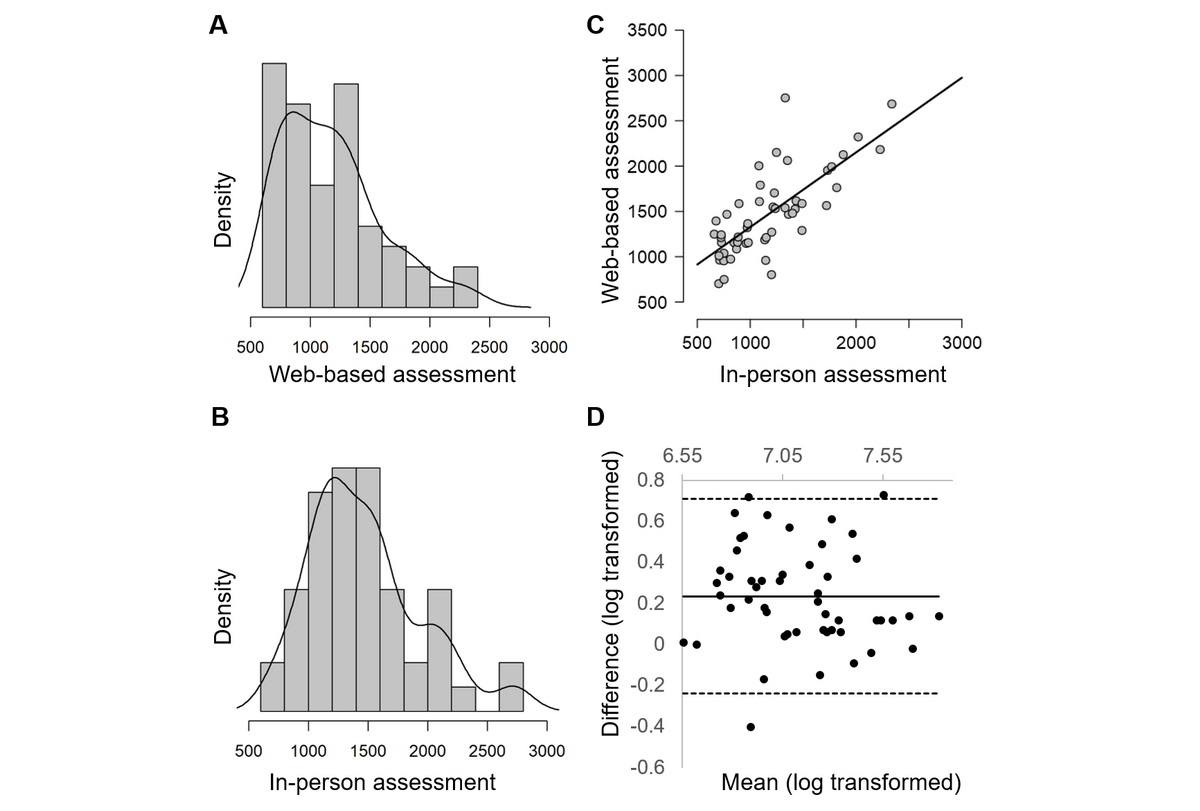
Comparability of Emotion Recognition Task median correct reaction time (in ms) across test settings. Density plot for (A) web-based assessment and (B) in-person assessment, showing broader distribution of timings (range 500-3000 ms) and slower overall timings for web-based assessment compared to in-person assessment (range 500-2500 ms); (C) scatterplot with reference line showing strong linear relationship between assessment settings (ρ=0.73); (D) Bland-Altman plot: mean difference (solid black line) is shifted above zero, demonstrating bias; dashed lines show limits of agreement. Comparable magnitudes of difference are seen throughout the range of measurements, and 94% of the data within limits of agreement.

For reaction time measures, Bland-Altman plots reflected bias in test settings in PRM-I and PRM-D response latencies and ERT Median Correct Reaction Time (eg, Figure 4), confirming the findings from the mixed model and Bayesian analyses. Additionally, for all reaction times, 94% of the data points were within limits of agreement, falling short of the 95% cutoff. Visual inspection of the plots confirmed comparable magnitudes of difference throughout the range of measurements, and regression analyses revealed no proportional bias (*R*^2^ range 0-0.05; *P*=.12 to .67).

## Discussion

This study examines the comparability of the widely used CANTAB administered unsupervised via the internet against a typical in-person lab-based assessment, using a counterbalanced within-subjects design. We imposed strict criteria for comparability, including satisfactory intersetting reliability, equivalence, and agreement across test settings. Overall, our results support the comparability of performance indices (errors, trials completed, and response sensitivity) acquired during web-based assessments. Reaction time measures show poorer comparability, with results revealing significant differences and poor agreement between test settings.

Bivariate correlation coefficients between the 2 modes of test administration ranged between 0.39 and 0.73, broadly in keeping with previous research comparing in-person and web-based assessment of other cognitive tasks [16,18,19]. The correlations reported here are similar to previously reported test-retest correlations in the CANTAB tests. An overview of test-retest correlations for CANTAB performance indices from previously published papers (and in different test populations) can be seen in Multimedia Appendix 1.

ICCs were higher for some tests than for others, with fair reliabilities (ICC ρ=0.40-0.49) seen for planning and executive function tasks (SWM Strategy and OTS performance measures). Previous research has shown that cognitive measures are subject to significant intraindividual variation [56]. A meta-analysis showed that test-retest reliabilities can differ depending on the tests completed and the cognitive functions that they tap into, with lower reliability typically seen for tests assessing executive functions and memory [57]. Poor reliability was seen for PRM-I percentage of correct trials in this study, which could be attributable to the low variance and high ceiling-level performance on this task in this healthy volunteer sample.

ICCs and Spearman correlations generally provided similar results, but showed greater discrepancy for reaction times, where there was a difference in the range and average between assessment settings. In these cases, ICCs typically presented a tempered correlation coefficient in comparison to Spearman correlations, reflecting that this statistic takes into account systematic error between assessments.

Learning effects are likely to have had an impact on concordance between test settings [16]. Practice effects with improvement on the second test administration were seen for 4 outcome measures (RVP A’, SWM Strategy, SWM Between Errors, and OTS Median Latency to Correct response). Previous work has shown increased susceptibility to specific tests, in particular those assessing visual memory, to practice effects [58]. The novelty of a test, particularly in the executive function domain, is also thought to influence susceptibility to practice effects [59]. Owing to these effects, it is recommended that a familiarization session, to reduce the immediate effect of novelty of tests and testing procedures, is used before baselining cognitive performance in clinical trials and other within-subject designs. Practice effects were not seen for the remaining outcome measures, which may be due to the use of alternate test stimuli [57]. In most CANTAB tests, stimuli are allocated at random from a broader stimulus pool during each assessment, reducing the likelihood that participants completed the same problems more than once.

Two out of 9 performance indices met all predefined criteria for comparability between measures. PAL Total Errors Adjusted and RVP A’ test scores did not differ between test settings, showed good intersetting reliability, and showed acceptable agreement on Bland-Altman plots. Additionally, for SWM Between Errors, Bland-Altman analyses were not completed, but the intersetting reliability was good, and there was no evidence of performance differences between settings. These measures are therefore determined to have good overall comparability vis-à-vis typical in-person assessment (overview shown in Table 3).

**Table 3:** The overall assessment of web-based outcome measures with regard to 3 criteria.

Outcome variable			Reliability^a^		Equivalence^b^		Agreement^c^
**Performance indices**							
	PAL^d^ total errors adjusted	✓^e^		✓		✓	
	PAL first attempt memory score	x^f^		✓		x	
	OTS^g^ problems solved on first choice	x		✓		✓	
	PRM-I^h^ percentage of correct trials	x		✓		—^i^	
	SWM^j^ between errors	✓		✓		—	
	SWM strategy	x		✓		x	
	ERT^k^ total hits	x		✓		✓	
	PRM-D^l^ percentage of correct trials	x		✓		—	
	RVP^m^ A’	✓		✓		✓	
**Reaction time measures**							
	OTS median latency to correct	x		✓		x	
	PRM-I median latency	✓		x		x	
	ERT median correct reaction time	✓		x		x	
	PRM-D median latency	x		x		x	
	RVP median latency	x		✓		x	

^a^: reliability criterion met where intraclass correlation coefficients ≥0.60.

^b^: equivalence criteria met where there is no significant difference between performance levels across test settings in mixed effects models, and data supporting the null hypothesis for Bayesian paired *t* tests).

^c^: agreement criteria met where ≥95% of data points lie within the limits of agreement on Bland-Altman plots, and there is no evidence of bias or proportional bias.

^d^PAL: paired associate learning.

^e^✓: criteria met.

^f^x: criteria not met.

^g^OTS: one touch stockings of Cambridge.

^h^PRM-I: pattern recognition memory immediate.

^i^—: analyses not completed.

^j^SWM: spatial working memory.

^k^ERT: emotion recognition task.

^l^PRM-D: pattern recognition memory delayed.

^m^RVP: rapid visual information processing.

Two additional performance indices were determined to have moderate comparability with respect to in-person assessment. The ERT Total Hits and OTS Problem Solved on First Choice outcome measures showed good equivalence and agreement, but below the threshold reliability indices. For the ERT Total Hits, the ICC fell just short of the imposed threshold (ICC coefficient=0.57).

Overall, none of the 5 web-based reaction time measures met more than one of the predefined comparability criteria, indicating that response latency measures are less easily translated from the lab to the home. Acceptable correlations between in-person and web-based assessments were undermined by a lack of equivalence and agreement between the measures. Correlation coefficients examine the linear relationship and relative consistency between 2 variables (the consistency of the position or rank of individuals in one assessment relative to the other [45]) rather than the absolute agreement between measurements within individuals [52,55], and are therefore insensitive to differences in metrics or variance (Figure 4).

Differences between settings could be due to a variety of factors. First, web-based assessments were completed on laptop and desktop computers that participants had readily available to them at home or elsewhere. Differences in computing equipment across settings are likely to have had an impact on response times [12]. Second, additional variance may have been introduced by distractions in the home environment, in comparison with the formal lab-based testing environment. We attempted to monitor and control for distraction and found that distraction more strongly affected reaction time measures during web-based testing. At the same time, all 5 outliers excluded during the current analyses were obtained during web-based assessments. Missing data from 2 participants was due to additional errors during web-based testing on the SWM task, which precluded the accurate calculation of test performance scores. Susceptibility to distraction and resultant increases in variance of test outcome measures are important to bear in mind when considering web-based testing as a substitute for, or in addition to, in-person testing.

### Limitations

The use of a healthy, relatively young, and highly educated sample may limit the generalization of findings to lesser-educated, clinical, or old-age samples. This research suggests that for the examined CANTAB performance indices, web-based assessments are likely to be a suitable alternative for similar samples. Further examination of the comparability of web-based assessment is now required in populations of clinical interest. In the longer-term, participants and patient groups with access restrictions may be the ones who benefit most from remote testing.

The study examined only the reliability of tests across settings and different devices, since all in-person tests were completed on touch screen iPads, and all web-based assessments on personal computers or laptops. Further research is required to examine whether reaction time data may be collected more consistently, where similar or the same devices are used across settings. Since the completion of this study, variance in workstation information is now routinely collected for CANTAB web-based tests, which allows for better determination of the effects of different workstations on test performance.

It is not clear how computer/device experience may have interacted with our results because we did not collect this information. However, our participants were recruited via Facebook, screened for inclusion online, and tested at home using their personal computing system, so it is likely that they had at least modest computer experience. Discrepancies between lab-based and web-based remote testing may be amplified for individuals with less computer experience, who may need to rely on the support of study staff to a greater extent.

The study was powered to detect moderate differences between test settings and was not adequately powered to identify subtle differences. Bayesian statistics were able to qualify the level of support for the null or alternate hypothesis, but much larger samples would be required to determine stronger evidence for the null hypothesis. Replication in a larger sample is now required to examine for the presence of any subtle differences between test settings.

Further work is now required to examine test-retest reliability for web-based assessments to identify whether test reliabilities are similar to those obtained during repeated in-person assessments. Our data show intersetting reliabilities, which are similar to previously reported test-retest reliabilities obtained during in-person assessments. Automated test scoring of performance indices, standardized across test administration and testing platforms, circumvent problems with rater-based variances in reliability. However, differences in computer hardware and software can impact reaction time data, and this must be borne in mind during web-based neuropsychological assessments.

### Overview and Implications

This study compared web-based CANTAB tests with gold-standard in-person administered lab-based assessments. Performance indices obtained in person showed broad equivalence, good agreement, and significant linear relationships with those obtained during web-based assessments. Overall, this study provides evidence for the comparability of a range of performance outcome indices examined using web-based testing in a healthy adult sample. Certain performance indices showed better comparability than others and should therefore be preferable for use where comparability with typical in-person assessment is needed. Reaction time indices were not found to be comparable, and greater care is required in the interpretation of web-based latency results in relation to typical in-person assessments.

## Appendix

Multimedia Appendix 1 Comparison of bivariate (Spearman) correlation of test performance between settings, with partial correlations which covary for elapsed time (in days) between assessments and test retest reliabilities of relevant CANTAB performance indices from previously published research.

## Abbreviations

CANTAB: Cambridge Neuropsychological Test Automated Battery
ERT: emotion recognition task
ICC: intraclass correlation
OTS: one touch stockings of Cambridge
PAL: paired associate learning
PRM: pattern recognition memory
RVP: rapid visual information processing
SWM: spatial working memory
