# Supplementary material for: Comparing Web-Based and Lab-Based Cognitive Assessment Using the Cambridge Neuropsychological Test Automated Battery: A Within-Subjects Counterbalanced Study
Source: J Med Internet Res. 2020 Aug 4;22(8):e16792. doi: 10.2196/16792 (PMC7435628; doi:10.2196/16792)
Supplement: Multimedia Appendix 1 [file jmir_v22i8e16792_app1.doc]

**Multimedia Appendix Table 1:** Comparison of bivariate (Spearman’s) correlation of test performance between settings, with partial correlations which covary for elapsed time (in days) between assessments.

| **Outcome variable** | **Spearman’s Correlation coefficient** | **Partial correlation coefficient** |
| --- | --- | --- |
| PAL Total Errors Adjusted | 0.54** | 0.54** |
| PAL Trials Correct on First Attempt | 0.45* | 0.45** |
| OTS Problems Solved on First Choice | 0.39* | 0.39* |
| OTS Median Latency to Correct | 0.53** | 0.54** |
| PRM-I Percentage of Correct Trials | 0.43* | 0.45* |
| PRM-I Median Latency | 0.59** | 0.57** |
| SWM Between Errors | 0.61** | 0.62** |
| SWM Strategy | 0.50** | 0.49** |
| ERT Total Hits | 0.54** | 0.53** |
| ERT Median Correct Reaction Time | 0.73** | 0.69** |
| PRM-D Percentage of Correct Trials | 0.49** | 0.51** |
| PRM-D Median Latency | 0.57** | 0.53** |
| RVP A’ | 0.71** | 0.70** |
| RVP Median Latency | 0.40* | 0.42* |

***P*≤.001, **P*≤.01, ^a^*P*=.05. Abbreviations: PAL – Paired Associate Learning, OTS – One-Touch Stockings of Cambridge, PRM-I – Pattern Recognition Memory Immediate, SWM – Spatial Working Memory, ERT – Emotion Recognition Task, PRM-D – Pattern Recognition Memory Delayed, RVP - Rapid Visual Information Processing

**Multimedia Appendix Table 2: Test retest reliabilities of relevant CANTAB performance indices from previously published research**. Note correlation coefficients represent Pearson’s or Spearman’s coefficients, unless specified as ICC where this corresponds to intra-class correlations.

| **Author name, year [reference]** | **Sample size and type** | **Mean age** | **Delay to retest** | **Test-retest correlations for performance outcomes** | | | | | | | | |
| --- | --- | --- | --- | --- | --- | --- | --- | --- | --- | --- | --- | --- |
|  |  |  |  | PAL Total Errors Adjusted | PAL First Attempt Memory Score | OTS Problems Solved on First Choice | PRM-I Percentage Correct | SWM Between Errors | SWM Strategy | ERT Total Hits | PRM-D Percentage Correct | RVP A’ |
| Karlsen et al. (2020) [1] | 75 healthy adults | 32 | 3 mo. | 0.73 | *NA* | *NA* | 0.60 | 0.71 | 0.79 | *NA* | 0.42 | 0.75 |
| Feinkohl et al. (2020) [2] | 45 older patients from outpatient clinics | 72 | 7 d. | *NA* | 0.72 *(ICC)* | *NA* | *NA* | *NA* | *NA* | *NA* | *NA* | *NA* |
|  |  |  | 3 mo. |  | 0.78 *(ICC)* |  |  |  |  |  |  |  |
| Cacciamani et al. (2018) [3] | 25 patients with mild cognitive impairment (MCI) | 68 | 6 mo. | 0.74 | *NA* | *NA* | 0.40 | 0.59 | 0.13 | *NA* | 0.48 | 0.55 |
|  |  |  | 12 mo. | 0.85 | *NA* | *NA* | 0.32 | 0.61 | 0.72 | *NA* | 0.22 | 0.76 |
| Gonclaves (2016) [4] | 34 healthy older people | 79 | 1 mo. | 0.68 | 0.56 | *NA* | *NA* | 0.85 | 0.67 | *NA* | *NA* | 0.71 |
| Lowe and Rabbitt (1998) [5] | 162 older volunteers | 70 | 4 wk | *NA* | 0.68 (*ICC)* | *NA* | *NA* | 0.68 *(ICC)* | *NA* | *NA* | *NA* | *NA* |
| Fowler (1995) [6] | 19 healthy volunteers | 59 | 1 mo. | 0.64 | *NA* | *NA* | *NA* | *NA* | *NA* | *NA* | *NA* | *NA* |
|  | 21 with questionable dementia | 58 |  | 0.71 |  |  |  |  |  |  |  |  |
|  | 16 with early Alzheimer’s dementia | 65 |  | 0.88 |  |  |  |  |  |  |  |  |

**References**

1. Karlsen RH, Karr JE, Saksvik SB, Lundervold AJ, Hjemdal O, Olsen A, Iverson GL, Skandsen T. Examining 3-month test-retest reliability and reliable change using the Cambridge Neuropsychological Test Automated Battery. Appl Neuropsychol [Internet] Routledge; 2020;0(0):1–9. [doi: 10.1080/23279095.2020.1722126]

2. Feinkohl I, Borchers F, Burkhardt S, Krampe H, Kraft A, Speidel S, Kant IMJ, Van Montfort SJT, Aarts E, Kruppa J, Slooter A, Winterer G, Pischon T, Spies C. Stability of neuropsychological test performance in older adults serving as normative controls for a study on postoperative cognitive dysfunction. BMC Res Notes [Internet] BioMed Central; 2020;13(1):3–8. [doi: 10.1186/s13104-020-4919-3]

3. Cacciamani F, Salvadori N, Eusebi P, Lisetti V, Luchetti E, Calabresi P, Parnetti L. Evidence of practice effect in CANTAB spatial working memory test in a cohort of patients with mild cognitive impairment. Appl Neuropsychol [Internet] Taylor & Francis; 2018;25(3):237–248. [doi: 10.1080/23279095.2017.1286346]

4. Gonçalves MM, Pinho MS, Simões MR. Test–retest reliability analysis of the Cambridge neuropsychological automated tests for the assessment of dementia in older people living in retirement homes. Appl Neuropsychol [Internet] Taylor & Francis; 2016;23(4):251–263. PMID:26574661

5. Lowe C, Rabbitt P. Test/re-test reliability of the CANTAB and ISPOCD neuropsychological batteries: Theoretical and practical issues. Neuropsychologia 1998;36(9):915–923. PMID:9740364

6. Fowler KS, Saling MM, Conway EL, Semple JM, Louis WJ. Computerized delayed matching to sample and paired associate performance in the early detection of dementia. Appl Neuropsychol 1995;2(2):72–78. [doi: 10.1207/s15324826an0202]
